# Gene Copy-Number Polymorphism Caused by Retrotransposition in Humans

**DOI:** 10.1371/journal.pgen.1003242

**Published:** 2013-01-24

**Authors:** Daniel R. Schrider, Fabio C. P. Navarro, Pedro A. F. Galante, Raphael B. Parmigiani, Anamaria A. Camargo, Matthew W. Hahn, Sandro J. de Souza

**Affiliations:** 1 Department of Biology and School of Informatics and Computing, Indiana University, Bloomington, Indiana, United States of America; 2 São Paulo Branch, Ludwig Institute for Cancer Research, São Paulo, Brazil; 3 Departamento de Bioquímica, Universidade de São Paulo, São Paulo, Brazil; 4 Centro de Oncologia Molecular–Hospital Sírio-Libanês, São Paulo, Brazil; 5 Brain Institute, Federal University of Rio Grande do Norte, Natal, Brazil; University of Washington, United States of America

## Abstract

The era of whole-genome sequencing has revealed that gene copy-number changes caused by duplication and deletion events have important evolutionary, functional, and phenotypic consequences. Recent studies have therefore focused on revealing the extent of variation in copy-number within natural populations of humans and other species. These studies have found a large number of copy-number variants (CNVs) in humans, many of which have been shown to have clinical or evolutionary importance. For the most part, these studies have failed to detect an important class of gene copy-number polymorphism: gene duplications caused by retrotransposition, which result in a new intron-less copy of the parental gene being inserted into a random location in the genome. Here we describe a computational approach leveraging next-generation sequence data to detect gene copy-number variants caused by retrotransposition (retroCNVs), and we report the first genome-wide analysis of these variants in humans. We find that retroCNVs account for a substantial fraction of gene copy-number differences between any two individuals. Moreover, we show that these variants may often result in expressed chimeric transcripts, underscoring their potential for the evolution of novel gene functions. By locating the insertion sites of these duplicates, we are able to show that retroCNVs have had an important role in recent human adaptation, and we also uncover evidence that positive selection may currently be driving multiple retroCNVs toward fixation. Together these findings imply that retroCNVs are an especially important class of polymorphism, and that future studies of copy-number variation should search for these variants in order to illuminate their potential evolutionary and functional relevance.

## Author Summary

Recent studies of human genetic variation have revealed that, in addition to differing at single nucleotide polymorphisms, individuals differ in copy-number at many regions of the genome. These copy-number variants (CNVs) are caused by duplication or deletion events and often affect functional sequences such as genes. Efforts to reveal the functional impact of CNVs have identified many variants increasing the risk of various disorders, and some that are adaptive. However, these studies mostly fail to detect gene duplications caused by retrotransposition, in which an mRNA transcript is reverse-transcribed and reinserted into the genome, yielding a new intron-less gene copy. Here we describe a method leveraging next-generation sequence data to accurately detect gene copy-number variants caused by retrotransposition, or retroCNVs, and apply this method to hundreds of whole-genome sequences from three different human subpopulations. We find that these variants account for a substantial number of gene copy-number differences between individuals, and that gene retrotransposition may often result in both deleterious and beneficial mutations. Indeed, we present evidence that two of these new gene duplications may be adaptive. These results imply that retroCNVs are an especially important class of CNV and should be included in future studies of human copy-number variation.

## Introduction

In recent years it has become apparent that changes in gene copy-number introduced by genomic duplication and deletion events are an important force driving adaptive evolution [1]. Examples of adaptive gene gains and losses have been found in a variety of organisms, including humans [2]–[4] and *Drosophila melanogaster* [5], [6]. Much attention has focused on gene duplications in particular, as they may facilitate the evolution of new gene functions [7], [8]. Given that all new gene duplicates must arise as polymorphisms, and the fact that genomic duplications and deletions can have negative phenotypic consequences [9]–[11], massive efforts have been made to identify regions of the genome differing in copy-number, referred to as copy-number variants (CNVs), among humans [2], [12]–[15] and other species (e.g., refs. [16]–[18]). These studies have revealed extensive copy-number variation especially within humans, with any two African individuals differing in copy-number at over 100 genes [2], [19].

It has been suggested that in humans the vast majority of gene duplications contributing to this variation result in a new copy located adjacent to the original gene [14]. However, a substantial number of new duplicates are inserted far from the original locus in humans and other mammals [20], [21], including genes duplicated by retrotransposition [22], [23]. These retrocopies, which are created when a messenger RNA transcript is reverse-transcribed and reinserted into a different location in the genome, are an especially interesting class of gene duplicate for several reasons. First, a new retrocopy will contain an entire coding sequence except when derived from an incomplete transcript. In addition, retrocopies occasionally carry promoter elements located downstream of the retrotranscribed transcript's transcription start site but located upstream of an alternative transcription start site [24]. Evidence that a substantial proportion of gene retrotransposition events result in functional gene copies, called retrogenes, come from both mammals [25], [26] and *Drosophila* [27]. In addition, patterns of gene movement onto and off of the X chromosome in mammals and off of the X in *D. melanogaster* suggest that many retrogenes are subject to positive selection (e.g., refs. [28]–[30]). Finally, processed pseudogenes, inactivated gene copies created by retrotransposition, have also been shown to influence expression levels of the parental gene copy, potentially disrupting its function [31], [32].

Despite the potentially important evolutionary and phenotypic consequences of retrogenes, current CNV-detection approaches are largely unable to find them. In fact, only one study of copy-number variation in humans was able to detect any polymorphic retrogenes [2]. Previously, we developed a method capable of leveraging next-generation sequence data to detect gene copy-number variants caused by retrotransposition, or retroCNVs, and used it to reveal that 13% of gene copy-number polymorphisms in *D. melanogaster* are caused by retrotransposition [30]. Although a similar method has been applied to detect retroCNVs in humans [33], there has been no detailed analysis of retroCNVs in humans to date. Here we apply an improved method to a number of sequenced human genomes, including data from the 1000 Genomes Project [34]. We find a surprising amount of variation due to retroCNVs within the human population—accounting for ∼12 genes differing in copy-number between any two individuals. By comparing retroCNV patterns to retrogene divergence, we reveal that retrotransposition is an important source of both adaptive and deleterious mutations in humans. We also find evidence that some of these retroCNVs may currently be under positive selection in humans. These findings underscore the functional and evolutionary importance of gene duplication via retrotransposition, and suggest that further study of retrogenes will illuminate the extent to which these retroCNVs affect human phenotypes and drive adaptive evolution.

## Results/Discussion

### RetroCNVS are common in human populations

**Figure 1:**
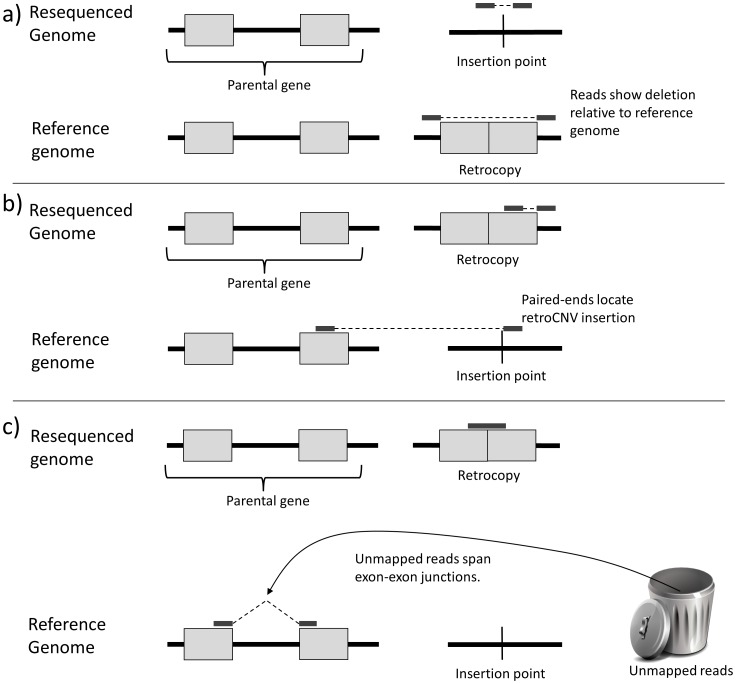
Detecting retroCNVs using sequence reads. a) RetroCNVs present in the reference genome are detected by searching for retrocopies in the reference that are absent from a sequenced individual, as revealed by paired-end reads spanning the location of the retroCNV and mapping too far apart from one another. b) RetroCNVs absent from the reference genome are detected by using paired-end reads to detect retroCNV insertion sites, and c) using reads that span exon-exon junctions but do not map to the reference genome.

In order to detect polymorphic retrocopies of protein coding genes segregating in human populations, we searched for evidence of retrocopy insertion sites using sequence reads from two human genomes that we sequenced ourselves with the SOLiD technology (denoted AAC and SJS), and additional genomes from the 1000 Genomes Project [34]. Briefly, this approach works by searching for paired-end reads spanning insertion sites of retrocopies present in the reference genome but absent from a resequenced genome (Figure 1a), or vice-versa (Figure 1b). We also searched low-coverage genomes resequenced for the 1000 Genomes Project [34] for exon-exon junction-spanning reads indicative of retroCNVs (Figure 1c), similar to our previous approach [30]. Because the whole genome must be searched in order to discover retroCNV insertions absent from the reference genome, such retroCNVs were initially discovered using a smaller set of 17 individuals (Table S1; Materials and Methods). These retroCNVs were then genotyped using paired-end sequence data from three subpopulations from the 1000 Genomes Project: 52 Yoruban individuals in Nigeria (referred to as the YRI subpopulation), 41 individuals of European ancestry in Utah (referred to as CEU), and 56 Han Chinese individuals and Japanese individuals from Tokyo (referred to as ASI). Because of this ascertainment scheme, these retroCNVs are expected to be biased towards higher frequencies than if they were discovered using the entire set of sequenced genomes. RetroCNVs present in the reference genome were identified using paired-end reads from all individuals sequenced for the 1000 Genomes Project, and are therefore unaffected by any ascertainment bias. We correct for this difference in ascertainment schemes where necessary in the analyses presented here. We find that our computational approach for retroCNV identification has high specificity and sensitivity, allowing us to estimate the contribution of retrotransposition to gene copy-number polymorphism in humans.

We identified 91 retroCNVs in total, finding that these polymorphisms account for 11.9 genes differing in copy-number between any two African individuals on average. Given that a recent comparison of pairs of individual human genomes has revealed gene copy-number differences at 105 genes on average (based on data from ref. [2]), our results suggest that retroCNVs could account for a sizable minority of human gene copy-number polymorphisms (although retroCNVs may often be non-functional). We were able to determine the insertion sites of 39 retroCNVs (18 present in the reference genome; 21 absent from the reference), and verify that retrocopy presence was the derived state for each of these (Materials and Methods); the remaining 52 retroCNVs were identified from reads spanning exon-exon junctions only and therefore have unknown insertion loci. While many of these retrocopies may contain only fragments of coding sequence, perhaps due to the low processivity of reverse-transcriptase or partial degradation of the mRNA used as template, we found that at least 41.8% (accounting for ∼6 complete gene copy-number differences between any two African genomes) of the retrocopies across all genomes are complete or near-complete retrogenes which may have the potential to be functional (see Materials and Methods).

To estimate the fraction of false positive retrogenes in our analysis, we attempted to validate all retroCNVs with known insertion sites by PCR amplification followed by sequencing. We confirmed 10 of 11 retroCNVs present in the reference genome (90.9%) that we were able to assay, and 17 of 21 (80.5%) retroCNVs absent from the reference genome. In the case of retroCNVs absent from the reference genome our experimental design does not allow us to differentiate between false positives and retroCNVs we could not amplify due to experimental difficulties such as low primer specificity (Materials and Methods), and most retroCNVs we could not amplify (whether present or absent in the reference) were flanked by repetitive elements. It therefore seems plausible that some or all of the four retroCNVs absent from the reference genome that we could not confirm are actually true positives. However, even if we conservatively assume that these four cases are false positives, our false positive rate across the set of 39 retroCNVs with known insertion loci is acceptably low (15.6%; validation results are listed in Table S2 and genomes used for validation are listed in Table S3). The remaining 52 retroCNVs may contain a higher fraction of false positives, and their relatively high fraction of singletons (67.3%) is consistent with this. However, we have previously shown that the exon-exon junction approach used to detect these retroCNVs is quite accurate [30]; thus, many of these 52 retroCNVs are likely true events, and the large number of singletons could in part be explained by somatic mutations in the cell lines used to obtain DNA for the individuals in the 1000 Genomes Project, in addition to false positives. In any case, the omission of these retroCNVs does not qualitatively affect any of the analyses described below. We estimate that the approach using paired-end reads to discover retroCNVs (whether present in or absent from the reference genome) was able to detect at least 77.4% of singleton retroCNVs inserted in non-repetitive sequence in the 17 discovery genomes. The false negative rate decreases dramatically for retroCNVs present more than once in the discovery set—we estimate that retroCNVs present in just two samples would be discovered ∼95% of the time (Materials and Methods). In addition, the exon-exon junction approach has previously been shown to be highly sensitive [30]; this implies that our dataset contains the vast majority of retroCNVs present in the genomes we examined during the discovery phase of our study. All retroCNVs included in our dataset, and their insertion coordinates when known, are listed in Table S2. The sets of genome sequences and retroCNVs included in each of our analyses are summarized in Table S4.

### Insertion patterns of retroCNVs

In contrast to tandem duplications caused by replication slippage, or sometimes by non-allelic homologous recombination (NAHR), retrotransposition results in a new gene duplicate located far from the parental copy. Unlike our previous examination of gene retrotransposition in *D. melanogaster* [30], in this study we were able to locate the insertion site of new retrocopies and therefore to examine precise patterns of gene movement caused by this type of duplication. Although there is an excess of fixed retrogene movements onto and off of the human and mouse X chromosomes relative to expectations [29], we do not see such a pattern in our set of retroCNVs (Table 1), suggesting differences in the contribution of adaptive evolution to polymorphic and fixed retrogenes. As we have previously done in *D. melanogaster*, here we conducted a statistical test for differences in patterns of movement between retroCNVs and fixed functional retrogenes. If gene movements onto and off of the X are neutral, then we expect the same proportion of such events among polymorphic retrocopies and fixed functional retrogenes; however, if movements involving the X chromosome are often adaptive, then we will observe a higher fraction of this class of movements among fixed retrogenes. We do in fact find a significantly higher fraction of fixed functional retrogenes than retroCNVs moving to and from the X chromosome (*P* = 0.0067; Fisher's exact test using fixed retrogene data from ref. [29]), lending further support to the hypothesis that natural selection is driving gene movement to and from mammalian X chromosomes [29]. This result remains significant when we only examine retroCNVs discovered in females (*P* = 0.0079), and is therefore not an artifact of reduced power to detect X-linked retroCNVs in males. Because retroCNVs absent from the reference genome were discovered using a different ascertainment scheme than retroCNVs present in the reference genome, combining them in this analysis could impact our results. However, this would only result in a deficit of retroCNVs moving to or from the X chromosome if such retroCNVs were more likely to be confined to lower allele frequencies by purifying selection than other retroCNVs, and there is no reason to expect such a difference in selective pressures. Moreover, after imposing the same ascertainment scheme on both retroCNVs present in and absent from the reference genome (Materials and Methods), we observe a similar but non-significant deficit of retroCNVs moving to or from the X (none of the 9 retroCNVs in this set involve movements to or from the X; *P* = 0.11). When we test separately for an excess of fixed functional retrogenes moving off of the X or moving onto the X, we do not see significance in either case (*P* = 0.150 for movements off of the X; Table S5; *P* = 0.0650 for movements onto the X; Table S6). However, although we have lower statistical power in these comparisons, we do observe trends suggestive of natural selection. Moreover, the excess of fixed functional retrogenes moving off of the X is significant when we compare retroCNVs to data from ref. [35] (*P* = 0.0077; Table S5); when we examine all retroCNVs, including those with an unknown insertion site, we also see a significant excess of fixed retrogenes originating on the X chromosome when comparing our data to both ref. [29] and ref. [35] (*P* = 0.032 and *P* = 3.6×10^−4^ respectively; Table S7). Combined with the observation that processed pseudogenes do not exhibit a bias of movement from the X [29], our data strongly suggest that natural selection is responsible for the excess of functional retrogenes moving off of the X chromosome in mammals, and perhaps onto the X chromosome as well. These observations could be the result of positive selection driving the fixation of new functional retrogenes moving to or from the X, selection to maintain such genes once they are established, or both of these mechanisms.

**Table 1:** RetroCNVs versus fixed retrogenes moving from an autosome to an autosome (A→A) from the X chromosome to the X (X→X), from the X to the autosomes (X→A), or vice-versa (A→X).

	RetroCNVs	Fixed retrogenes*
A→A or X→X	36	70
A→X or X→A	3	29

* Data from Emerson et al. [29].

While it is widely believed that gene duplicates created by retrotransposition are almost always dead-on-arrival pseudogenes because they do not carry all regulatory elements from the parental copy with them, it has been shown that a retrocopy inserted into another gene will often exploit that gene's regulatory machinery in order to be expressed [26]. We therefore examined the insertion point of our retroCNVs to determine how many were inserted into existing genes. We found that over one-half (20 of 39) of retroCNVs were inserted into genes, with all but one of these retroCNVs being inserted into an intron (Table S2). This does not represent a significant deviation from what one would expect if retrocopy insertions were distributed uniformly across the genome, as introns make up roughly 40% of the human genome (*P* = 0.60; χ^2^ test). Although there does not appear to be a strong bias in polymorphism data, we compared retroCNVs to the 7,831 retrocopies (functional or otherwise) identified in the reference genome (Materials and Methods), nearly all of which are fixed, and found a deficit of fixed human retrocopies in introns compared to retroCNVs: 50.0% of retroCNVs versus 31.8% of fixed retrocopies are found in introns (Table 2; *P* = 0.022; Fisher's exact test; *P* = 0.012 using fixed retrocopies from ref. [26] with *d_S_*<0.1 when compared to their parent gene). Again, similar to the reasoning laid out above, this implies that retrocopies inserted into introns are often deleterious, as was suggested by Vinckenbosch et al. [26]. Indeed, the results in Table 2 suggest that roughly one-half of intronic retrocopy insertions are eliminated by purifying selection. A similar deficit of fixed intronic retrocopies is observed when we impose the same ascertainment scheme on all retroCNVs, as described in Materials and Methods (62.5% of retroCNVs found in introns versus 31.8% of fixed retrocopies), although this comparison is no longer significant (*P* = 0.12), perhaps in part due to diminished statistical power. Because this is a comparison of patterns of retroCNVs that may not be functional to fixed retrocopies that are mostly pseudogenes, the simplest interpretation of this result is that the insertion of retrocopies into genes may often be deleterious even when the inserted retrocopy is non-functional. Thus, intronic insertions may often be deleterious regardless of the content of the inserted sequence. This interpretation is supported by the observation that tandem duplications occurring within introns are often subject to purifying selection in *Drosophila* [17].

**Table 2:** RetroCNVs versus fixed retrocopies inserted in intronic versus intergenic sequence.

	RetroCNVs	Fixed retrocopies
Intronic insertions	19	2,492
Intergenic insertions	19	5,339

If the above interpretation is correct, then it could imply that roughly half of the genic retroCNVs we detect here are deleterious and would not be allowed by selection to reach fixation. This interpretation is substantiated by the lower allele frequencies of intronic versus intergenic retroCNVs when examining only retroCNVs present in the reference genome (avg. frequency in YRI is 0.46 for intronic and 0.72 for intergenic retroCNVs; *P* = 0.75) or absent from the reference genome (0.11 for intronic versus 0.16 for intergenic; *P* = 0.95). We performed this comparison separately for retroCNVs present and absent from the reference genome in order to control for ascertainment bias, as these retroCNVs had different ascertainment schemes. While these differences are not significant, they are consistent with selection acting against intronic insertions, especially given evidence that non-retroCNV insertions within introns are often deleterious as discussed above. Consistent with this interpretation, it has been noted that fixed retrocopy insertions are less likely to be intronic than expected if retrocopies are inserted with uniform probability across the genome [26], although there is evidence of an insertion bias associated with chromatin accessibility in *Drosophila* [36]. Overall, there is substantial evidence that insertions of retrocopies or other sequence into introns are often deleterious.

Since one would presume that retrocopies inserted into introns are also more likely to be expressed, our results suggest that retrotransposition could be an important source of new functional gene copies as well as potentially deleterious mutations. An additional possible functional consequence of the insertion of retroCNVs into introns is the formation of sense-antisense pairs, as we previously suggested [37]. Consistent with this possibility, we find that 10 of 20 retrocopies inserted into another gene are on that gene's minus strand (Table S2). We also find that one retroCNV, a copy of RPL3, switches strands mid-sequence, most likely due to 5′ inversion during retrotransposition [38].

### Segregating chimeric genes created by retrotransposition

Another interesting consequence of the insertion of a retrocopy into an intron of a host gene is the possibility of chimeric transcription of the host and the retrocopy. Chimeric genes are likely an important source of new gene functions [39], and the large fraction of retroCNVs inserted into introns suggests that retrotransposition could be an important source of these genes. Indeed, there are several known cases of retrotransposition resulting in functional chimeric genes in humans [40], [41] and *Drosophila* [6], [42], [43], with some of these genes showing evidence for adaptive evolution [6], [44].

In order to search for evidence of chimeric transcripts among the 20 retroCNVs inserted within existing genes, we examined RNA-seq data from lymphoblast tissues from 60 HapMap individuals of European descent [45]. We found that 20% (4 of 20) of these retroCNVs show evidence of chimeric expression. The chimeric transcript *CBX3*-*C15orf57*, where the *CBX3* retroCNV is inserted in-between the second and third exons of *C15orf57*, shows evidence of expression as a chimera in 20 individuals. The chimeric combination *SDHC*-*RPA1* forms a sense-antisense pair, with *SDHC* inserted in-between the fifth and sixth exon of *RPA1*; the chimeric transcript is expressed in 6 individuals. *UQCR10*-*C1orf194*, in which *UQCR10* is inserted into the second exon of *C1orf194* is expressed in a single individual. An examination of the sequencing read confirming the validity of this retroCNV reveals that the *UQCR10* portion of this transcript is not in proper reading frame. The *RPL18A*-*TXNRD1* combination, in which *RPL18A* is inserted in-between the third and fourth exons of *TXNRD1*, was also found to be expressed in one individual. We also found evidence of chimeric transcripts derived from SKA3-DDX10 in a breast cancer cell line and in a lymphoid cell line (HCC1954 and HCC1954-BL from ref. [46]), both derived from an individual genotyped for *SKA3*. The *SKA3* retroCNV is inserted in-between the tenth and eleventh exons of *DDX10*, forming a sense-antisense pair.

Because three of these chimeric transcripts involve either a sense-antisense pair or the retroCNV apparently being inserted out of reading frame, they may be nonfunctional and perhaps deleterious. Alternatively, it has been suggested that chimeric transcripts could result in novel protein coding regions even if they are not in sense-sense orientation or proper reading frame [25]. In addition, we have only examined expression data for chimeric transcripts from lymphoblast cell lines for the majority of our retroCNVs, and two additional cell lines for a single retroCNV (*SKA3*; Materials and Methods), and may therefore be underestimating the number of segregating chimeric genes caused by the incorporation of retroCNVs into existing genes. While further work is required to determine the number of these new genes and their functional consequences, our results suggest that retrotransposition could be a source of evolutionary novelty creating not only new gene duplicates but new genes with potentially novel functions.

### Evidence that positive selection may be acting on retroCNVs

**Figure 2:**
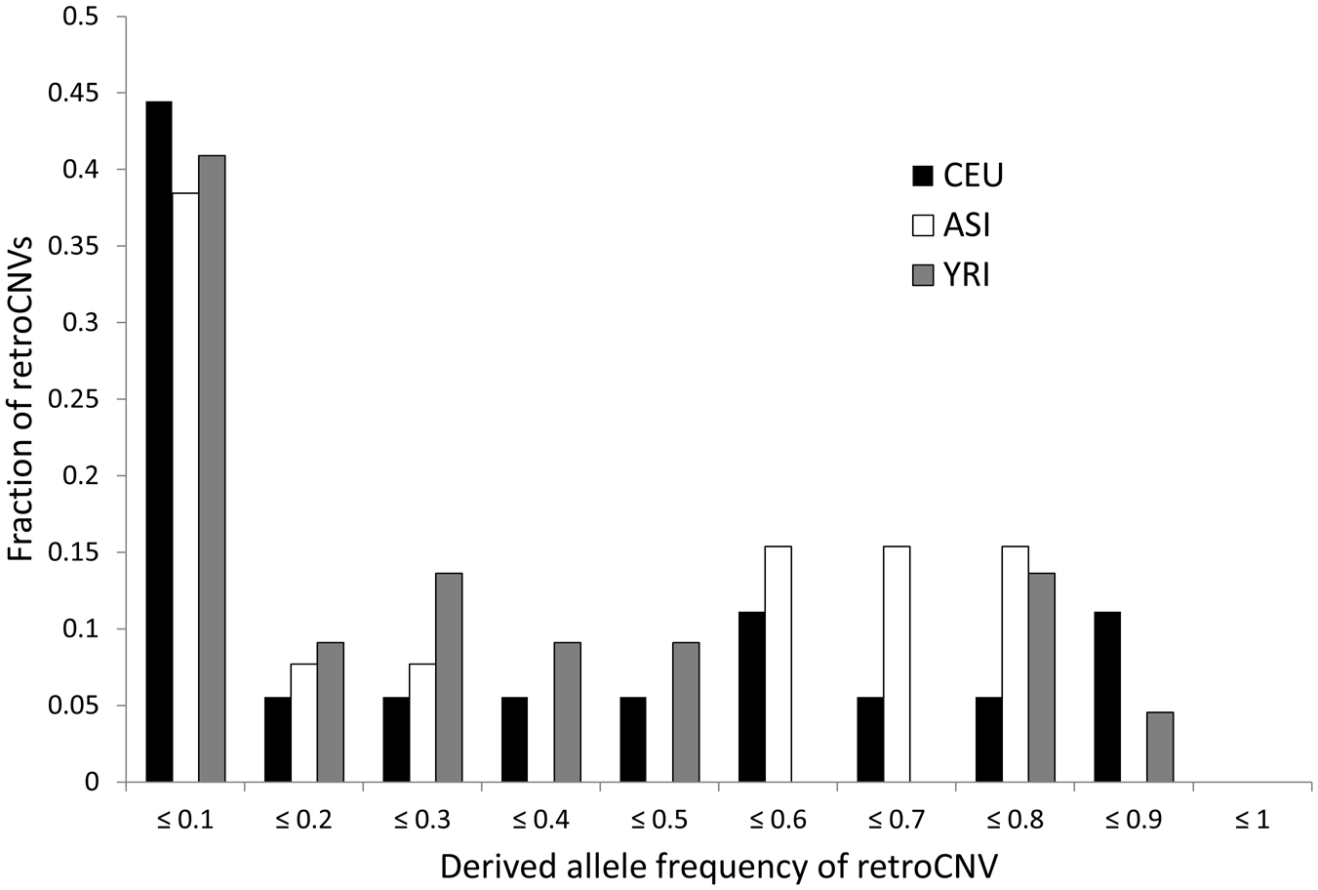
Estimated derived allele frequencies of retroCNVs segregating in three human subpopulations. Allele frequencies were calculated as described in the Materials and Methods. RetroCNVs fixed in or absent from a given subpopulation are not shown.

In order to examine the population dynamics of retroCNVs, we used both insertion presence/absence information at retroCNV insertions and evidence of retrotransposition from exon-exon junction-spanning reads to genotype 39 retroCNVs whose insertions we were able to locate. After estimating allele frequencies for these retroCNVs in three human populations (Materials and Methods), we noticed that several had very high derived-allele frequencies (Figure 2; frequencies listed in Table S2). While this observation is consistent with positive selection driving retroCNVs to fixation, the fact that many of our retroCNVs were ascertained in a sample of 17 genomes (AAC, SJS, and 15 individuals from the 1000 Genomes Project) biases our frequency spectra towards higher frequency variants. We therefore searched for more direct evidence of adaptive natural selection acting on individual retroCNVs. Although previous genome-wide studies of copy-number variation have searched for evidence of natural selection sweeping duplications towards fixation [2], [14], these searches were conducted at regions containing the parental copy and not necessarily the daughter copy. This was because location of the daughter locus was not known, and was simply assumed to be proximate to the parental locus. These approaches would therefore fail to detect evidence of positive selection on dispersed duplications, a limitation that does not affect our analysis because we have identified the exact location of the new duplicates. Conversely, if the insertion sites of duplicates are not known, many previous studies of ongoing selective sweeps in humans [47], [48] may have detected the signature of positive selection on an inserted sequence that was not known to lie in the selected region.

In addition to examining the correct locus, testing for adaptive evolution requires accurate genotyping. We therefore genotyped all 39 retroCNVs with known insertion sites as homozygous for retroCNV presence, heterozygous, or homozygous absent using our short-read sequences. In order to assess our genotyping accuracy, we initially compared our genotyping results for the retroCNV of *DHFR* to those of Conrad et al. [2], who were able to genotype this retroCNV as well. We found that our genotypes agreed for 100% of individuals genotyped as homozygous for retroCNV presence by Conrad et al., for 85% of individuals genotyped as heterozygous, and for 98% of individuals genotyped as homozygous absent. Because Conrad et al. [2] may have committed genotyping errors as well, these percentages can be thought of as a lower bound on our genotyping accuracy, suggesting that our genotyping is highly accurate. In order to gain additional confidence in our genotyping accuracy, we analyzed the genotypes of two available trios from the 1000 Genomes Project, finding that no analyzed retroCNVs violated Mendelian inheritance (Table S8), although these genomes had higher coverage than the rest of our data set. In addition, we experimentally validated the genotypes of *DHFR* and *GNG10* (discussed below) in 36 individuals (Table S3) and found that our genotyping is also accurate in genomes with lower coverage, with 94.4% and 91.7% of genotyping calls confirmed for these two retroCNVs, respectively. At these two retroCNVs we correctly genotyped 85.3% of heterozygous individuals and 100% of homozygotes, similar to our results in comparison to those of Conrad et al. [2].

The action of positive selection on an allele results in a rapid increase in the frequency of the haplotype containing the selected allele in the population. The swift nature of this rise in frequency results in a decrease in genetic diversity among chromosomes containing the selected allele compared to neutral expectations. We therefore examined nucleotide diversity (π) in regions flanking retroCNV insertions, finding several retroCNVs with a marked reduction in diversity among haplotypes containing the retroCNV relative to the other haplotypes in the population (Materials and Methods). However, a deficit of diversity is expected among haplotypes sharing a derived allele regardless of its selective importance [49]. With this in mind, we used coalescent simulations [50] to ask whether the ratio of π among haplotypes containing a retroCNV to π among haplotypes lacking it, which we refer to as π_der_/π_anc_, was lower than expected under neutrality (Materials and Methods). This is similar to the haplotype-based test first suggested by Hudson et al. [51], the sole difference being that we contrast π between the derived and ancestral allelic classes, rather than the number of segregating sites. For a polymorphism segregating in the absence of selection, we expect the observed ratio of π_der_/π_anc_ to be typical when compared to those generated from the neutral coalescent for derived alleles of the same sample frequency. For a polymorphism sweeping to fixation, on the other hand, relatively little diversity is expected among chromosomes containing the selected allele that is rapidly rising in frequency, and this allelic class would therefore exhibit a lower π_der_/π_anc_ ratio than polymorphisms of the same frequency simulated under neutrality.

**Figure 3:**
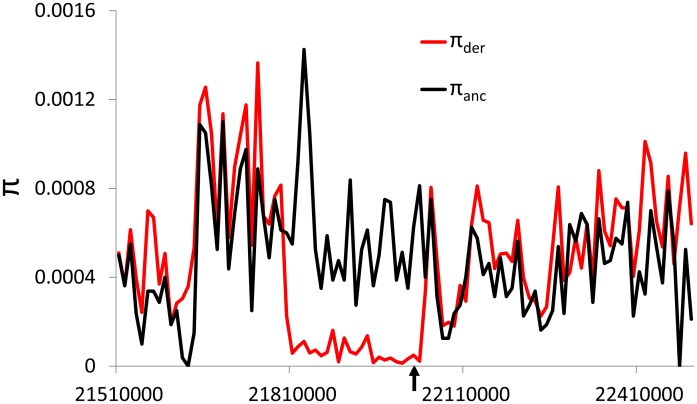
Reduced nucleotide diversity on chromosome 18 among chromosomes containing the *DHFR* retroCNV in CEU. π is shown in 10 kilobase windows for chromosomes containing the *DHFR* retroCNV (red) and those lacking this retroCNV (black). The location of the retroCNV insertion is marked by an arrow. While there is little difference in nucleotide diversity distal to the retroCNV, there is a recombination hotspot in that region (data from ref. [65]).

We were able to perform this test on 17 retroCNVs in the CEU subpopulation, 16 in YRI, and 13 in ASI (Materials and Methods). Two retrocopies are candidates for positive selection according to this test: the retrocopy of *DHFR* appears to be experiencing positive selection in individuals of European descent (*P* = 0.0083; Figure 3), as does a retrocopy of *GNG10* in both Europeans (*P* = 0.0094; Figure S1) and Africans (*P*<1.1×10^−4^; Figure S2). If we correct for multiple testing by conservatively assuming that all 46 tests for selection that we conducted were independent—even though many tests were of the same retroCNVs but in different subpopulations—the false discovery rate (FDR) for the *DHFR* and *GNG10* retroCNVs in Europeans is 0.14, while the FDR for the *GNG10* retroCNV is 0.0051 in Africans. As stated above, a deficit of diversity is expected within haplotypes containing a new mutation under the neutral coalescent. However, this deficit is less pronounced for polymorphisms with relatively high derived-allele frequencies such as the *DHFR* and *GNG10* retroCNVs because the amount of diversity associated with any allele is proportional to its frequency. The reductions in heterozygosity shown in Figure 3, Figure S1, and Figure S2 may therefore be suggestive of positive selection; this interpretation is supported by the results of our coalescent-based test that takes allele frequency into account.

The *DHFR* retroCNV, previously discovered by Anagnou et al. [52], is inserted into the fourth intron of *PSM8*, forming a sense-antisense pair. The ORF of this retrocopy perfectly matches that of the parental copy of *DHFR* in the reference genome [53]. *DHFR* codes for dihydrofolate reductase, deficiency of which causes megaloblastic anemia and neurological disease [54], and is required for nucleotide synthesis [55]. *DHFR* has an important role in cell growth, and its inhibition has been used in antibacterial [56] and antitumor drugs [57]. This retrocopy also exhibited a similar reduction in nucleotide diversity in the Asian subpopulation, although this pattern was not significant by our test (*P* = 0.099; Figure S3). *GNG10*, which has been associated with melanoma [58], has a retrocopy that forms a sense-sense pair with *SBF2*, which has been implicated in Charcot-Marie-Tooth disease [59]. To gain further confidence in these results, we compared the π_der_/π_anc_ ratios observed for these candidates to those calculated from random regions flanking SNPs with similar derived allele frequencies, finding that relatively few SNPs in the human genome exhibited lower π_der_/π_anc_ ratios than these retroCNVs, even though some of these loci are likely themselves under positive selection. For example, just 2.5% and 5.5% of loci in the genome exhibited lower ratios of π_der_/π_anc_ than the *DHFR* retroCNV in Europeans and the *GNG10* retroCNV in Africans, respectively (Materials and Methods).

Although we experimentally determined that our genotype calls at these two retroCNVs were quite accurate, genotyping error could still affect the analyses described above. We therefore conducted a further test based on integrated haplotype scores (iHS), a statistic designed to detect extended haplotypes characteristic of ongoing sweeps, around these two retroCNV insertions [48]. Importantly, this test is not dependent on our genotype assignments. We find that only 1.2% of random genomic regions exhibit stronger biases toward extreme iHS values than the region containing the *GNG10* retroCNV in Africans, the strongest candidate identified by our coalescent-based test (Materials and Methods). Additionally, only 5.7% of random genomic regions exhibit more extreme iHS values than the *DHFR* retrocopy in Asians, where we observed a suggestive but non-significant signal of selection in our coalescent-based test. We cannot know with certainty that natural selection is responsible for the patterns of diversity around these two retroCNVs, or that the retroCNVs themselves rather than polymorphisms in linkage disequilibrium with them are the targets of any such selection. Nonetheless, our findings that the haplotypes containing these retroCNVs exhibit reduced diversity and reside within regions identified by an extended haplotype test suggest that these retroCNVs should be considered candidates for adaptive natural selection. This evidence that multiple retroCNVs currently segregating in human subpopulations could potentially confer an increase in fitness suggests that retrotransposition could be an important source of adaptive alleles in humans.

### Conclusions

Given the evolutionary significance of gene retrotransposition in humans and other species, we sought to examine the extent of gene copy-number variation caused by retroCNVs in human subpopulations. This effort resulted in the first set of gene duplication polymorphisms caused by retrotransposition in humans obtained from next-generation sequence data. Experimental validation shows that our methodology has high sensitivity and precision. These data reveal that retroCNVs are quite common, accounting for roughly a dozen gene copy-number differences between any two African genomes on average. Our data also provide direct evidence that gene retrotransposition events are often adaptive. First, a comparison of retroCNV insertion patterns with fixed retrogenes supports the hypothesis that the excess of retrogenes moving onto and off of the X chromosome during mammalian evolution is driven by natural selection [29]. Moreover, our high genotyping accuracy combined with our ability to locate the insertion sites of many common retroCNVs allowed us to detect signatures of natural selection acting on these variants. We find evidence that at least two retroCNVs detected in this study may be affected by adaptive natural selection. Indeed, because we may not have perfect power to detect all polymorphisms under positive selection, we may be underestimating the fraction of retroCNVs undergoing selective sweeps. This result implies that retrotransposition could be an important force driving ongoing human adaptation.

We also find that many retroCNVs are inserted into the introns of existing genes. While we find that these retroCNVs are less likely to reach fixation than intergenically inserted retrocopies and may therefore often be deleterious, these retroCNVs are more likely to be expressed [26]. Moreover, five particularly interesting cases of this type of retroCNV result in a chimeric transcript consisting of sequence from the retroCNVs and the gene in which it was inserted. Given that chimeric genes can have important functional consequences [44], and that we are very likely underestimating the fraction of chimeras among retroCNVs, retrotransposition could be an important source of chimeric proteins with the potential to perform novel functions. Taken together, these results imply that gene retrotransposition has been and may continue to be an important source of adaptive alleles in humans, and could be an underappreciated source of mutations with negative phenotypic consequences as well.

## Materials and Methods

### Data sources

The human genome reference sequence (hg19/GRCh37) was downloaded from the UCSC Genome Browser (http://genome.ucsc.edu/). Gene models and transcript sequences of protein-coding genes were downloaded from version 57 of Ensembl [60]. Human reference mRNA sequences were downloaded from NCBI Reference Sequence project (http://www.ncbi.nlm.nih.gov/RefSeq/). Alignments, raw sequences, and unmapped reads from resequenced whole genomes were obtained from the 1000 Genomes Project (ftp://ftp-trace.ncbi.nih.gov/1000genomes/). We also sequenced two individual human genomes using the SOLiD3 platform; DNA samples from these individuals were donated to the Tumor Bank from the Hospital Alemão Oswaldo Cruz in São Paulo, Brazil after informed consent was obtained. These sequences were aligned to the reference genome using the mapping/pairing pipeline from BioScope (v3.1; http://www.solidbioscope.com/) with default parameters. The sets of individual genomes and retroCNVs examined in each phase of our analysis are listed in Table S4. Additionally, RNA-seq (paired-end) data from 60 HapMap individuals [45] were searched for evidence of chimeric transcripts.

### Sequencing two individual human genomes

The two individuals sequenced here (AAC and SJS) filled out consent forms and donated DNA to the Tumor Bank from the Hospital Alemão Oswaldo Cruz; this databank was approved by the Hospital's Institutional Review Board. Twenty micrograms of genomic DNA were sheared using HydroShear to generate fragments with an average size of 2.0 kb. DNA fragments were then repaired to generate blunt ends and ligated to adaptors. DNA fragments of 2–3 kb were size-selected in agarose gels and subsequently circularized by ligation of a biotinylated internal adaptor. After removing non-circularized fragments, circularized DNA was treated with DNA polymerase I for nick-translation, followed by digestion with T7 exonuclease and S1 nuclease, which generated tags longer than 50 bp from the adaptor edges. Digested products were ligated with P1 and P2 adaptors, purified and amplified with 12 PCR cycles. A total of 96 picograms of the resulting library were then used for emulsion PCR. Approximately 300 million beads from each library were deposited on one slide, followed by 50 bp mate-pair sequencing on a SOLiD3 instrument, according to the manufacturer's protocol.

### Identification of retroCNVs present in the reference genome

In order to detect retroCNVs present in the human reference genome, we first identified retrocopies present in the reference using a pipeline consisting of four steps: i) We aligned all human RefSeq transcripts to the human genome reference sequence; ii) All alignments overlapping multi-exon genes or the gene of the transcript's origin were removed. iii) Intronless alignments containing at least two exons from the parental gene, and exons mapped adjacently (without gaps) were selected; iv) Finally, we grouped sequences mapping to the same genomic region and removed putative retrocopies appearing to arise from genomic duplication. Using this approach, we found 7,831 retrocopies, which is similar to the number found in other databases, such as pseudogene.org (www.pseudogene.org) and Hoppsigen (http://pbil.univ-lyon1.fr/databases/hoppsigen).

In order to determine whether any of these 7,831 were retroCNVs segregating in humans, we downloaded alignments for all individuals from the 1000 Genomes Project that had whole-genome paired-end data and examined paired-end reads lying within 5 kb of a retrocopy. Paired-end reads that mapped further apart from one another than expected (indicative of a deletion) and that spanned a retrocopy without overlapping it were kept as evidence of a retroCNV (Figure 1a). Putative retroCNVs spanned by more than five paired-end reads were examined, and those not appearing to be artifacts of misalignment were subjected to experimental validation.

### Identification of retroCNVs absent from the reference genome using reads at insertion sites

In order to detect retroCNVs not present in the human reference genome we examined paired-end read alignments from 15 individuals from the 1000 Genomes Project (Table S1), including two high-coverage parent-offspring trios. Examining these genomes and the genomes of AAC and SJS, we searched for paired-ends with one read mapped entirely within exonic sequence of a known gene (the putative parental gene) and the other read mapped to a distinct genomic region: i.e. on a different chromosome or on the same chromosome with a mapping distance higher than the average insert size of the paired-end library (a putative retroCNV insertion site; Figure 1b). We then removed insertion sites located within 2 kb of known retrocopies as they may represent alignment artifacts, insertion points overlapping retrotransposons (defined by RepeatMasker), and insertion sites supported by five or fewer non-redundant paired-end reads mapping to exonic regions of a single parental gene. All 39 candidates containing an insertion site were manually curated to remove those resulting from alignment artifacts, and subjected to experimental validation (for details, see “Experimental validation of retroCNVs” below).

### Identification of retroCNVs absent from the reference genome using reads from exon–exon junctions

In order to search for additional retrotransposition events in low-coverage human genomes, we aligned unmapped reads from low-coverage genomes from the 1000 Genomes Project (the same genomes from ref. [34] used in the genotyping step described below) to human transcript sequences using BWA with default parameters (similar to the approach described in ref. [30]). Only Illumina and 454 reads were included in this analysis, as we noticed that the shorter SOLiD reads used in the 1000 Genomes Project introduced an extremely high number of false positives. Reads mapping across exon-exon junctions within these transcripts were taken as initial evidence of retrotransposition (Figure 1c). In particular, a gene was considered retrotransposed if there was i) at least one read in at least one individual spanning an exon-exon junction with at least 10 bp of the read crossing the junction, or ii) at least two distinct reads with different sequences (whether in the same individual or not) with at least 5 bp crossing an exon-exon junction. We only considered alignments having no more than 4% mismatches, and no more than 0.2*min(r,l) mismatches, where r and l are the number of bases in the read mapping to the left and right sides of the exon-exon junction, respectively. We used BLAT [61] to search for exon junction sequences (20 bp on either side of the junction) and to determine which of these junctions had partial or complete matches in the reference genome with the potential to introduce false positives. We removed from the analysis junctions with a BLAT hit in the reference genome with at least 90% identity and 10 bp on either side of the junction mapping to the reference genome. BLAT hits spanning the junction by less than 10 bp were kept in the analysis, but the number of base pairs spanning the junction was added to the mapping cutoffs required for calling retrogenes as described above. For example, if an exon-exon junction mapped to the reference genome with 7 bp of the match spanning the junction, two reads would need at least 12 bp spanning the junction, or one read would need at least 17 bp spanning the junction in order to call a retroCNV. All aligments reporting a putative retrotransposed gene were examined manually and reproduced using BLAT, and alignments that could be explained by reasons other than a retrotransposition event (e.g. reads mapping to the reference genome with a few mismatches) were removed.

In order to find the insertion site of retroCNVs identified using the exon-exon junction approach, all alignments for each of the individuals with whole-genome sequences from the 1000 Genomes Project were downloaded and paired-end reads with one read mapped to the 5′ or 3′ exon of a putative parental gene were extracted. Since genome coverage for most of these individuals is low, we merged all reads from these individuals and then selected insertion sites supported by more than five paired-end reads summing across individuals. For this analysis we have also excluded: i) insertion sites related to two or more parental genes; ii) insertion sites located within 2 kb of known retrocopies; iii) and insertion points overlapping retrotransposons. Insertion sites were manually curated in order to remove those resulting from misalignment.

### Controlling for different ascertainment schemes

RetroCNVs present in and absent from the reference genome have different ascertainment schemes, with retroCNVs present in the reference genome discovered by examining all sequenced individuals in our data set and retroCNVs absent from the reference discovered in a smaller discovery set, or from exon-exon junction spanning reads (Table S4). Ascertainment bias could therefore affect observed patterns of retroCNV insertions when these two sets of retroCNVs are combined. We therefore repeated our comparisons of fixed and polymorphic retrocopies with respect to X versus autosomes and introns versus intergenic regions after imposing the same ascertainment scheme on both retroCNVs present in and absent from the reference. This ascertainment scheme required a retroCNV to have support for the non-reference allele from more than five read-pairs in at least one of the 17 discovery genomes (Table S1), and ignored evidence from exon-exon junction spanning reads. Note that this ascertainment scheme is more stringent for both retroCNVs present in and absent from the reference genome, and therefore the number of retroCNVs discovered is reduced substantially. When comparing allele frequencies of intronic and intergenic retroCNVs, we simply performed the analysis separately for retroCNVs present in the reference genome and retroCNVs absent from the reference genome, thereby preventing differences in ascertainment from affecting the results. The results of our coalescent-based tests for selection are not affected by ascertainment bias as each test is conditioned on the observed allele frequency of the retroCNV being tested.

### Genotyping retroCNVs in human populations

We performed *in silico* genotyping for our complete set of retroCNVs identified using all three methods: from the reference genome absent in sequenced individuals, from paired-ends supporting insertion sites absent from the reference genome, and from exon-exon junction-spanning reads. These retroCNVs were genotyped in CEU (n = 41 unrelated individuals with paired-end data), YRI (n = 52), and ASI (CHB+JPT; n = 56) individuals with Illumina paired-end sequence data generated for the 1000 Genomes Project [34]. Genotyping proceeded as follows: for the set of retroCNVs present in the reference genome, we searched for paired-end reads for which one read mapped to the retroCNV itself and the other read mapped to the genomic region flanking the retroCNV (evidence of retroCNV presence). We also searched for paired-end reads spanning (without overlapping) the retroCNV regions (evidence of retroCNV absence). For the set of retroCNVs not present in the reference genome, we searched for paired-end reads for which one read mapped to the exonic region of a parental gene and the other read mapped to the insertion point of the retroCNV (evidence of retroCNV presence). We also searched for paired-end reads mapping to both sides of the insertion point and presenting the expected distance and orientation (evidence of retroCNVs absence). Heterozyogous individuals were identified as those exhibiting evidence for both retroCNV presence and absence. Reads spanning exon-exon junctions by 5 bp (plus any additional bases required due to partial matches of the exon junction in the reference genome as described above) were also used for determining whether a retroCNV was present in a given individual. For each of these strategies only one supporting read or read-pair was required for genotyping. For one gene, *CACNA1B*, heterozygotes could not reliably be distinguished from homozygotes. Allele frequencies were calculated for this retroCNV from the fraction of individuals with the presence allele (whether heterozygous or homozygous), in the same manner as the other 38 retroCNVs for which the insertion was located (see below). This retroCNV was omitted from tests for positive selection.

### Assessing the completeness of retroCNV sequences

RetroCNVs were considered complete or nearly complete if the retrocopy contained at least part of the 5′-most and 3′-most exons in the retroposed transcript. For retroCNVs present in the reference genome, we simply examined the sequence of the retrocopy. For retroCNVs absent from the reference genome, all isoforms of the parental gene that could potentially have been reverse-transcribed given the exons known to be present in the retrocopy from exon-exon junction-spanning reads and read-pairs mapping to insertion sites were examined.

### Estimating allele frequencies of retroCNVs

Because low coverage may cause our genotyping approach to undercall heterozygotes, and because we cannot distinguish homozygotes from heterozygotes using exon-exon junctions, we estimated the fraction of individuals containing each retroCNV (whether homozygous or heterozygous). This fraction, f, was calculated as the number of individuals with evidence of a retroCNV divided by the total number of individuals with evidence of either presence or absence of the retroCNV. We then estimated allele frequencies by assuming Hardy-Weinberg equilibrium: if f is the fraction of individuals with the retroCNV, f = p^2^+2pq, and 1−f = q^2^. Therefore, q = (1−f)^1/2^ and p = 1−(1−f)^1/2^. Note that retroCNVs with very high allele frequencies (i.e., with no individuals homozygous absent) will be incorrectly estimated as having an allele frequency of 1 although they are truly polymorphic with *p* approaching 1. Because we could not detect evidence of absence for retroCNVs with no detected insertion sites, we restricted allele frequency analyses to the 39 retroCNVs for which we could locate the insertion. These frequency estimates were used to compare allele frequencies of intronic and intergenic retroCNV insertions. Because exon-exon junction-spanning reads can produce evidence of retroCNV presence but not absence, potentially biasing allele frequency estimates, we repeated this comparison after omitting these data and verified that this bias did not qualitatively affect our results. In order to estimate the number of pairwise differences in retroCNV copy-number in the YRI subpopulation, we included retroCNVs genotyped by exon-exon junction spanning reads only, treating individuals with no evidence of retroCNV presence as homozygous absent, and calculating f as above, then estimating p and q and taking the summation of 2pq for each retroCNV.

Although it seems unlikely that any of these retroCNVs are caused by deletions of genes recently retrotransposed, we nonetheless polarized each of the 39 retroCNVs with a known insertion locus by using BLAT [61] to search for a retrocopy in the syntenic location of the chimpanzee genome as identified by liftOver [62]. Using this approach we confirmed that the presence of the retrocopy was indeed the derived allele for each of these 39 retroCNVs.

### Experimental validation of retroCNVs

We attempted to validate all 39 retroCNVs with known insertion sites via PCR and DNA sequencing. For retroCNVs not present in the reference genome we designed primer pairs with one matching the parental gene sequence and one matching the insertion site sequence; this will yield a PCR product only when the retroCNV is present. We therefore cannot differentiate between false positives and cases where we could not amplify due to experimental difficulties. Indeed, two retroCNVs we attempted to amplify, *CACNA1B* and *FOXK2*, yielded numerous PCR products of different sizes and may lie within regions difficult to amplify with specificity and may not necessarily be false positives. Nonetheless, we conservatively report a false positive rate that assumes retroCNVs absent from the reference genome and yielding no clear PCR product are false positives. For retroCNVs present in the reference genome, we designed primers spanning the daughter (i.e. newly inserted) copy. In this case, both true and false positives should yield PCR products, and the sequence of the product is used to distinguish true positives from false positives. Thus, false positives are not confused with PCR failures. For larger retroCNVs, it is possible that primer pair spanning the insertion site may not reliably amplify across the retrocopy. In such cases, we designed an additional primer pair with one primer within the retrocopy and one primer in the flanking insertion sequence to identify retroCNV presence, while the primer pair spanning the insertion site was used to identify retroCNV absence. Primers for PCR were designed based on the reference genome sequence (hg19/GRCh37) using the Primer3 [63] and Oligotech (Oligos Etc., Eugene, OR) software packages. PCR reactions were carried out in a 25 µL reaction containing 50 ng of genomic DNA, 1× Taq DNA polymerase buffer (Invitrogen), 0.1 mM dNTP, 1 mM MgCl2, 1 unit Taq DNA polymerase (Invitrogen) and 6 pmol of each forward and reverse primer. Amplification conditions were: initial denaturation for 4 min at 94°C followed by 35 cycles of 45 sec at 94°C, 45 sec at 58°C and 1 min at 72°C and a final extension of 10 min at 72°C. PCR products were analyzed on 1% agarose gels and sequenced using the Big Dye Terminator kit (Applied Biosystems) and an ABI3100 Prism sequencer. The sequenced product was then examined to determine if it was consistent with the validation status indicated by the presence and/or size of the PCR product. The genomes used to validate these retroCNVs are listed in Table S3. These same genomes and methods were used to validate genotype calls for the *GNG10* and *DHFR* retroCNVs, using DNA from genomes listed in Table S3. DNA samples from all of these genomes were obtained from the Coriell Cell Repository (http://ccr.coriell.org).

### Identification of chimeric transcripts containing retroCNVs

In order to detect chimeric expression of retroCNVs we downloaded paired-end alignments of RNA-Seq data from 60 European individuals (including 39 of the 41 Europeans in our data set) from ref. [45] and searched for read-pairs with unambiguous alignments where one read mapped to an exon of the retroCNV's parent gene (or the retrocopy itself if present in the reference genome) and the other read mapped to an exon of the gene in which the retroCNV was inserted. Only chimeric transcripts supported by 5 reads or more were considered, and only retroCNVs inserted into a known gene were included in this analysis.

We also tested for the expression of a chimeric transcript formed by the SKA3 retroCNV and its host gene, DDX10, using a pair of primers designed in SKA3 (5′ TCCCTCAGAAAAAGCTATGGTG 3′) and in DDX10 (5′ TCAAGGAGAGTGATGATTC 3′). Total RNA was extracted using Trizol following the manufacturers' instructions (Invitrogen) and RNA integrity was analyzed using agarose gels. Reverse transcription was carried out using the Superscript III First Strand Synthesis Kit (Invitrogen). RT-PCR reactions were carried out in a 25 µl reaction mixture containing 1 µl of cDNA, 2.5 µl Taq DNA polymerase buffer, 0.1 mM dNTPs, 6.0 pmol of each, 1.0 mM MgCl2, and 1 U Taq DNA polymerase (Invitrogen). PCR conditions were as follows: 4 min at 94°C (initial denaturation), 35 cycles of 45 s at 94°C, 45 s at 58°C, and 1 min at 72°C, with a final extension step of 10 min at 72°C. RT-PCR products were analyzed on 8% silver-stained polyacrylamide gels. Sequencing reactions were carried out using DYEnamic (ET Terminator Cycle Sequencing Kit, Amersham Pharmacia) and an ABI 3130XL sequencer (Applied Biosystems). This experiment was performed in four cell lines: two from a single individual previously genotyped for the SKA3 retrogene [46], and two negative controls.

### Estimating the false-negative rate of retroCNV discovery using paired-ends

In order to estimate an upper bound on the fraction of retroCNVs that we could not discover in the 17 genomes from the discovery set using paired-ends (AAC, SJS, and 15 individuals from the 1000 Genomes Project), we examined 10 fixed retrocopies present in the reference genome. Since these retrocopies are always homozygous present, we doubled the number of required read-pairs in order to detect a retroCNV as present (simulating the discovery of a heterozygous retroCNV). From these data we estimate the fraction of singletons (retroCNVs present in one of the 17 genomes, or 1/34 chromosomes, examined to discover retroCNVs with this method) our approach would fail to detect—a conservative upper bound on our false negative rate. This fraction can be used to estimate the fraction of retroCNVs present in i chromosomes in our discovery set by simply raising it to the i^th^ power.

### Searching for positive selection around retroCNV insertions

In order to test for positive selection acting on retroCNVs, we first downloaded SNP genotype data for all SNPs within 100 kb of the insertion point for each retroCNV segregating in the CEU, YRI, and ASI subpopulations. Next, we inferred the haplotypic phase of each of these retroCNVs and their flanking SNPs by running fastPhase [64] with default parameters. RetroCNV genotype data from insertion sites were included as fastPhase input, with modifications in two cases involving retroCNVs absent from the reference genome. First, if a retroCNV was genotyped as homozygous absent in an individual from insertion site-spanning paired-end reads, but exon-exon junction spanning-read data from that same individual supported the presence of the retroCNV, the genotype was set to heterozygous for retroCNV presence. Second, if no paired-end reads were available for genotyping an individual and exon-exon junction data supported retroCNV presence, the individual was genotyped as having the retroCNV on one chromosome, and as having an unknown genotype on the other.

By examining the position homologous to insertion sites in the chimpanzee genome, we found that all of our insertions were derived. Our test for selection then asks whether there is a significantly lower value of π, the average number of pairwise differences per site, within the set of haplotypes having the retroCNV (π_derived_) compared to the set of haplotypes lacking the retroCNV (π_ancestral_), controlling for differences in allele frequencies [51]. We took the ratio of these measures, which we refer to as π_der_/π_anc_, as our test statistic. In order to determine if there was less nucleotide diversity in the set of haplotypes containing the retroCNV than is expected under neutrality, we performed 10,000 coalescent simulations using ms [50] with the same number of polymorphisms observed within 100 kb on either side of the retroCNV (plus one additional polymorphism taking the place of the retroCNV), and the same number of chromosomes as in the real sample. For these simulations, we assumed a single, flat recombination rate given by the region flanking the retroCNV insertions, as estimated from HapMap Phase II data [65]. For the CEU and ASI populations, a demographic model involving a bottleneck was used (using ms parameters -eN 0.05 0.5 -eN 0.15 1.5), and for YRI a recent population expansion was used (-eN 0.0 1.5). We then examined whether there was any polymorphism within the medial 25% of the simulated region having the same derived allele frequency as the retroCNV such that the ratio of π within haplotypes containing the derived allele to π within haplotypes containing the ancestral allele was less than or equal to the ratio calculated by partitioning the observed data according to alleles at the retroCNV. We calculated the P-value as the fraction of these simulated polymorphims meeting this criterion. This test was performed for each retroCNV segregating in each subpopulation in which at least two chromosomes contained the retroCNV and two chromosomes lacked it. We were able to test 17 retroCNVs in the CEU subpopulation, 16 in YRI, and 13 in ASI.

In order to determine whether candidate retroCNVs identified by this approach were also outliers compared to other polymorphisms segregating in humans, we compared the observed π_der_/π_anc_ ratios to those calculated from non-overlapping 200 kb windows of SNPs from the 1000 Genomes data (http://www.1000genomes.org/). For each 200 kb window in each population, we calculated π_der_/π_anc_ for up to one SNP lying within 10 kb of the center of the window and having a derived allele frequency landing in the same 5% bin as that of the retroCNV. We then calculated the fraction of these SNPs having π_der_/π_anc_ less than or equal to that of the retroCNV for candidates for positive selection.

As an alternative method to search for evidence of positive selection in regions containing retroCNVs, we downloaded integrated haplotype scores (iHS) from ref. [48] and compared the density of high-|iHS| SNPs in regions containing retroCNVs to random genomic regions. Regions with a high density of high-|iHS| SNPs have previously been used as evidence of positive selection [48]. High-|iHS| SNPs were defined as those with iHS scores within either the upper or lower 2.5% tail of the empirical distribution of iHS scores from that same population. Within the retroCNV region, extended by 50 kb on each side, we counted the fraction of SNPs with high |iHS|, and calculated a χ^2^ statistic comparing this fraction to the 0.05 expectation. We then repeated this test within 10,000 genomic regions of the same size, counting the fraction of these regions with a higher χ^2^ statistic than in the retroCNV region.

## Supporting Information

Figure S1 Nucleotide diversity on chromosome 11 among chromosomes containing and lacking the *GNG10* retroCNV in CEU. π is shown in 10 kilobase windows for chromosomes containing the *GNG10* retroCNV (red) and those lacking this retroCNV (black). The location of the retroCNV insertion is marked by an arrow. As with *DHFR*, there is a recombination hotspot distal to the retroCNV (data from ref. [65]).
(TIF)

Figure S2 Nucleotide diversity on chromosome 11 among chromosomes containing and lacking the *GNG10* retroCNV in YRI. π is shown in 10 kilobase windows for chromosomes containing the *GNG10* retroCNV (red) and those lacking this retroCNV (black).
(TIF)

Figure S3 Nucleotide diversity on chromosome 18 among chromosomes containing and lacking the *DHFR* retroCNV in ASI. π is shown in 10 kilobase windows for chromosomes containing the *DHFR* retroCNV (red) and those lacking this retroCNV (black).
(TIF)

Table S1 Genomes used to discover retroCNVs absent from the reference genome.
(XLS)

Table S2 Coordinates of retrotransposed genes and their insertion sites (hg19).
(XLS)

Table S3 Genomes used for experimental validation.
(XLS)

Table S4 RetroCNVs and genome sequences examined in each analysis.
(XLS)

Table S5 Movements of retroCNVs and fixed retrogenes originating on the X chromosome and originating on the autosomes.
(XLS)

Table S6 Movements of retroCNVs and fixed retrogenes to the X chromosome and to the autosomes.
(XLS)

Table S7 Movements of retroCNVs and fixed retrogenes originating on the X chromosome and originating on the autosomes, including retroCNVs with an unknown insertion site.
(XLS)

Table S8 Genotypes of two parent-offspring trios.
(XLS)
