# Supplementary figures and images for: Gene Copy-Number Polymorphism Caused by Retrotransposition in Humans
Source: PLoS Genet. 2013 Jan 24;9(1):e1003242. doi: 10.1371/journal.pgen.1003242 (PMC3554589; doi:10.1371/journal.pgen.1003242)

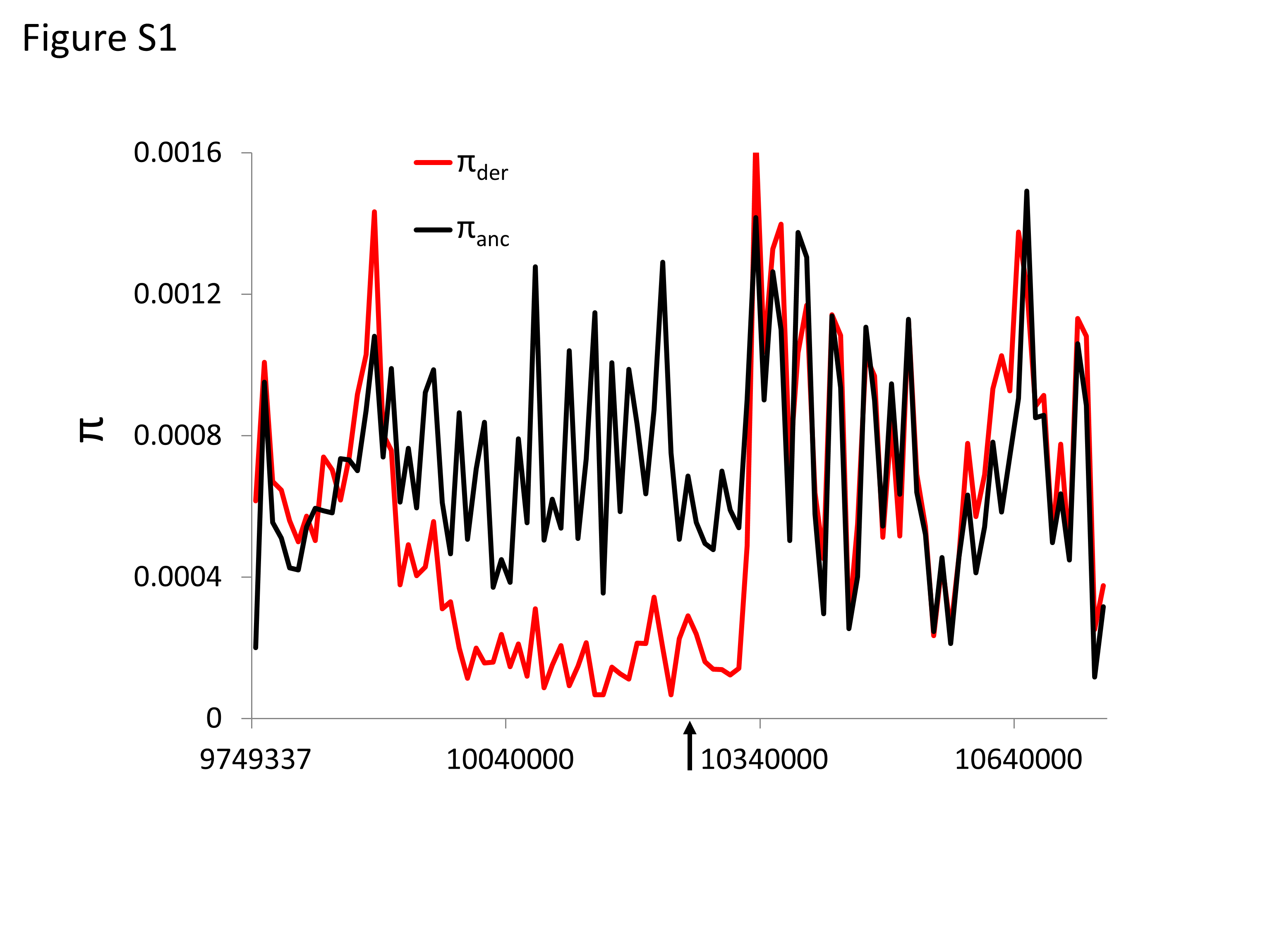

Supplement: Figure S1 — Nucleotide diversity on chromosome 11 among chromosomes containing and lacking the GNG10 retroCNV in CEU. π is shown in 10 kilobase windows for chromosomes containing the GNG10 retroCNV (red) and those lacking this retroCNV (black). The location of the retroCNV insertion is marked by an arrow. As with DHFR, there is a recombination hotspot distal to the retroCNV (data from ref. [65]). (TIF) [file pgen.1003242.s001.tif]

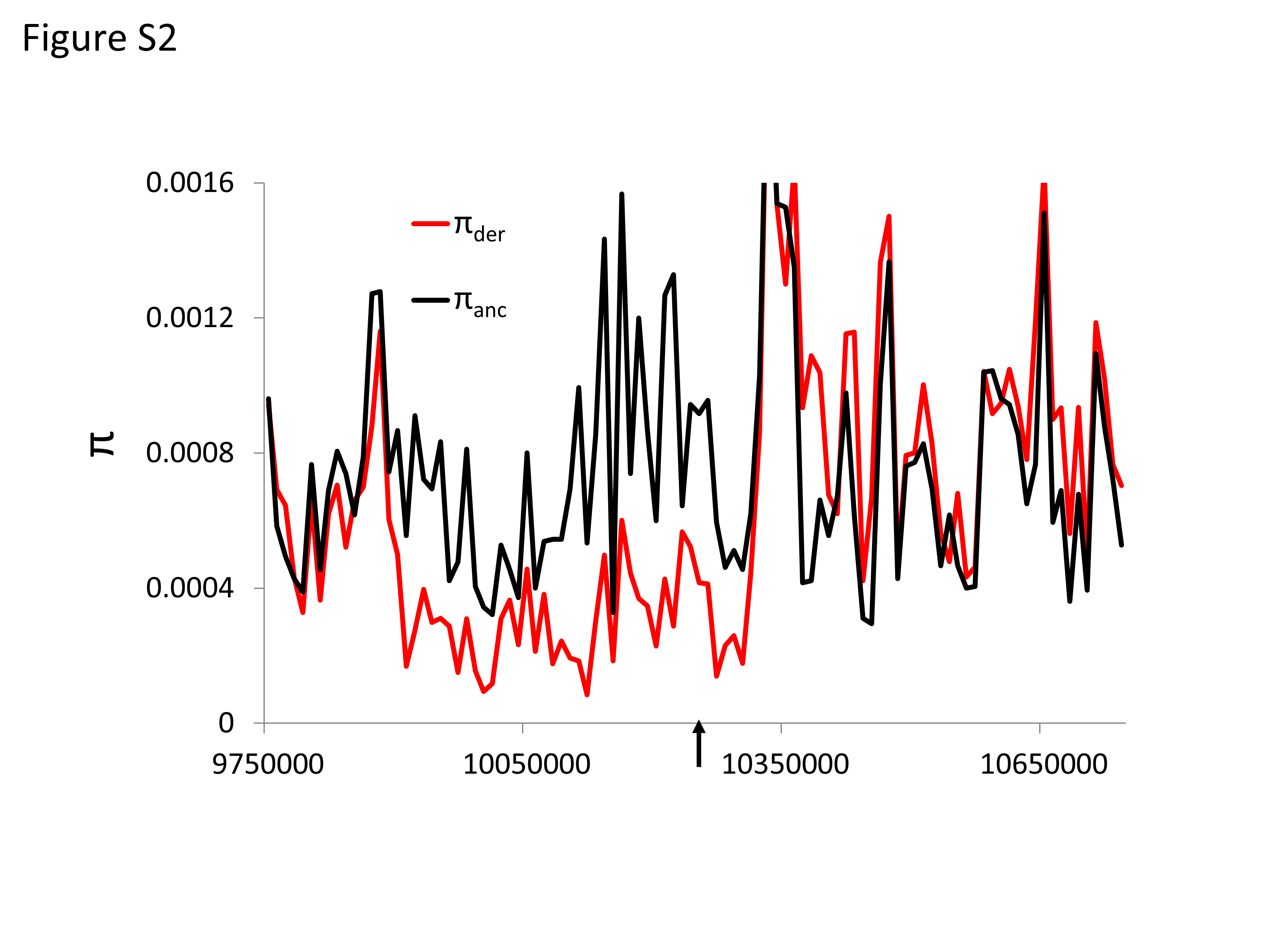

Supplement: Figure S2 — Nucleotide diversity on chromosome 11 among chromosomes containing and lacking the GNG10 retroCNV in YRI. π is shown in 10 kilobase windows for chromosomes containing the GNG10 retroCNV (red) and those lacking this retroCNV (black). (TIF) [file pgen.1003242.s002.tif]

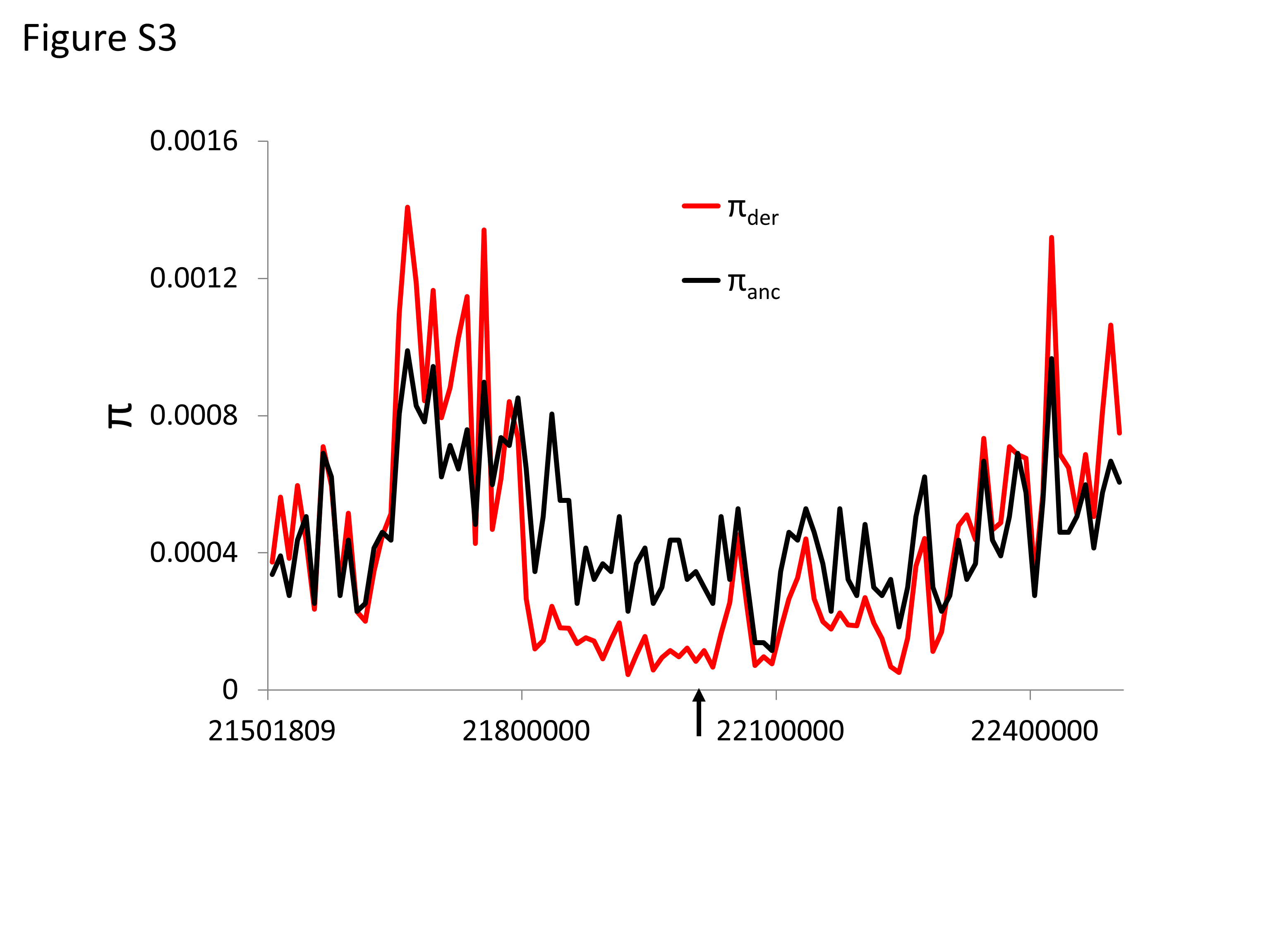

Supplement: Figure S3 — Nucleotide diversity on chromosome 18 among chromosomes containing and lacking the DHFR retroCNV in ASI. π is shown in 10 kilobase windows for chromosomes containing the DHFR retroCNV (red) and those lacking this retroCNV (black). (TIF) [file pgen.1003242.s003.tif]
